# New anti‐tau VHH: from biophysical characterization to proof‐of‐concept in experimental models

**DOI:** 10.1002/alz.088152

**Published:** 2025-01-09

**Authors:** Clément Danis, Raphaelle Caillierez, Justine Mortelecque, Séverine Bégard, Orgeta Zejneli, Jean‐Christophe Rain, Morvane Colin, Elian Dupre, Isabelle Landrieu, Luc Buee

**Affiliations:** ^1^ Inserm, LilNCog, Lille, Hauts de France France; ^2^ CNRS, Integrative Structural Biology, Lille, Hauts de France France; ^3^ Hybrigenics Service, Hybribody, Paris, Paris France; ^4^ Université de Lille, Lille, Hauts‐de‐France France

## Abstract

**Background:**

Tau proteins aggregate in a number of neurodegenerative disorders known as tauopathies. Various studies have highlighted the role of microtubule‐binding domains in the intracellular aggregation of Tau protein.

**Method:**

Using a library of synthetic VHHs humanized in collaboration with Hybrigenics, we have developed a number of anti‐tau VHHs. We use a combination of biophysical and biochemical methods, cell‐based assays, viral vectors and tau transgenic mice to explore the ability of these VHHs to target intracellular tau protein.

**Result:**

A dozen anti‐tau VHHs were obtained near and in microtubule‐binding regions. After screening and optimization, three of them (F8‐2, H3‐2 and Z70) were selected for further studies and AAV vector delivery to tau transgenic mice. Biochemical characterization of F8‐2 has already been published [1]. VHH Z70 targeting the PHF6 epitope has already demonstrated its ability to reduce tau seeding in cellular FRET assays and by lentiviral vectorization in animals [2]. Here, using AAV vectors, we demonstrate its safety in wild‐type mice and its efficacy in tau transgenic mice. VHH H3‐2 has a unique mode of action which will be discussed.

**Conclusion:**

In our tau pipeline, we have developed innovative new therapeutic tools, which are at various stages of development for preclinical validation.

**References**

[1] Danis C, et al. (2022) Inhibition of Tau seeding by targeting Tau nucleation core within neurons with a single domain antibody fragment. **Mol Ther**, 30(4):1484‐1499

[2] Dupré E, et al. (2019) Single chain antibody fragments as new tools for studying the neuronal Tau protein physiopathology. **ACS Chemical Neurosci**, 10(9):3997‐4006

